# Glucose and Fatty Acid Metabolism in a 3 Tissue In-Vitro Model Challenged with Normo- and Hyperglycaemia

**DOI:** 10.1371/journal.pone.0034704

**Published:** 2012-04-11

**Authors:** Elisabetta Iori, Bruna Vinci, Ellen Murphy, Maria Cristina Marescotti, Angelo Avogaro, Arti Ahluwalia

**Affiliations:** 1 Division of Metabolic Diseases, Department of Clinical and Experimental Medicine, University of Padua, Padua, Italy; 2 Centro Interdipartimentale di Ricerca ″E.Piaggio″, University of Pisa, Pisa, Italy; 3 CNR Institute of Clinical Physiology, Pisa, Italy; University of Tor Vergata, Italy

## Abstract

Nutrient balance in the human body is maintained through systemic signaling between different cells and tissues. Breaking down this circuitry to its most basic elements and reconstructing the metabolic network in-vitro provides a systematic method to gain a better understanding of how cross-talk between the organs contributes to the whole body metabolic profile and of the specific role of each different cell type. To this end, a 3-way connected culture of hepatocytes, adipose tissue and endothelial cells representing a simplified model of energetic substrate metabolism in the visceral region was developed. The 3-way culture was shown to maintain glucose and fatty acid homeostasis in-vitro. Subsequently it was challenged with insulin and high glucose concentrations to simulate hyperglycaemia. The aim was to study the capacity of the 3-way culture to maintain or restore normal circulating glucose concentrations in response to insulin and to investigate the effects these conditions on other metabolites involved in glucose and lipid metabolism. The results show that the system’s metabolic profile changes dramatically in the presence of high concentrations of glucose, and that these changes are modulated by the presence of insulin. Furthermore, we observed an increase in E-selectin levels in hyperglycaemic conditions and increased IL-6 concentrations in insulin-free-hyperglycaemic conditions, indicating, respectively, endothelial injury and proinflammatory stress in the challenged 3-way system.

## Introduction

The metabolic profile of healthy individuals is well known. For example, after an overnight fast, healthy subjects retain normal circulating glucose concentrations, high circulating FFAs and glycerol, and low lactate levels [1]. After feeding, their glucose shows a slight rise, which quickly returns to normal levels, circulating FFA and glycerol concentrations drop, and lactate rises. In obese and diabetic patients, whole body metabolism is deranged such that normal metabolic patterns are altered [2]–[5]. The distorted metabolic profiles of individuals with metabolic diseases have been the subject of intensive in-vivo investigations on humans and animals as well as on simple in-vitro monocultures. It is clear from in-vivo studies that an organism’s nutritional status is communicated through metabolic signaling by adipose tissue and the liver and perceived by all organs [6]. Nutritional overload is characterized by alterations in metabolic profiles and impairments in insulin responsiveness. In-vitro studies have provided a great deal of information on individual molecular pathways in single cells, but cannot be used to investigate how cross-talk between different tissues determines whole body metabolism. Indeed, while the signaling mechanisms responsible for homeostasis, inflammation and injury have been well studied at the local cellular level, it is less clear how alterations in one cell or tissue are communicated to other parts of the body.

A number of key organs in different locations interact to maintain the systemic energy balance. They are linked by the vascular network; a vital communication highway for metabolic signaling between tissues. Besides hormonal signaling, interaction between tissues is also mediated by the metabolites themselves, even in the absence of insulin and glucagon [7]–[10]. Clearly, a better understanding of the metabolic cross-talk among different organs is essential in order to find the most appropriate interventions to treat or prevent metabolic diseases.

To probe cross-talk between tissues and determine how it may contribute to the whole body metabolic profile, we have designed a modular bioreactor system to systematically reconstruct endogenous metabolism in-vitro [11], [12]. The system consists of interconnected chambers, each of which houses a specific tissue or organ of relevance to energetic substrate metabolism. The chambers are linked together by the flow of a common medium, much as the bloodstream connects different tissues or organs in the body. By increasing the number of interactions and variables step-wise in a properly scaled model, nutrient dynamics between organs and their contribution to systemic metabolism can be investigated. Thus, breaking down the metabolic circuitry to its most basic elements and reconstructing the network in-vitro, the specific contribution of each tissue in maintaining the energy balance can be assessed. In addition, analysis of metabolic interactions among different cell types can be used to gain insights about the ways that different tissues are relevant in determining the overall metabolic profile and explore how systemic signaling of nutrient balance is maintained, or how it may be disrupted.

To reduce the complexity of the system, hepatocytes, adipose tissue and endothelial cells were the first building blocks used to assemble an in-vitro metabolic model representing the central abdomen. These 3 elements constitute a relevant portion of the abdominal viscera and are amongst the first to sense the presence of nutritional input after ingestion. The liver has a central role in energetic substrate metabolism, and its multiple metabolic functions are carried out by hepatocytes. It processes, converts and substantially regulates all 3 energetic substrates: fats, sugars and proteins. Hepatocytes are therefore a key element in any in-vitro metabolic system. Adipose tissue is a key regulator of energy related cross-talk in the body. and is fundamental to the modulation of insulin sensitivity in skeletal and hepatic tissue [13]–[15]. Far from being a passive fat storage depot, adipose tissue integrates and outputs information on the overall nutritional status in the body, messaging distant organs through its impressive array of signaling molecules such as TNF-α (tumor necrosis factor-α), IL-6 (interleukin-6), adiponectin, leptin and lipoprotein lipases. Over-nutrition is in fact associated with a state of chronic low grade adipose tissue inflammation, which induces vascular dysfunction and a consequent series of knock-on effects in other organs and tissues [16], [17]. The two are connected together by the vascular system, which acts as an important modulator of metabolic signaling. Indeed the endothelium is one of the first tissues to perceive the presence of excess nutrient intake [6] as well as the first to contact nutrients as they spill into the blood stream after digestion. Other organs involved in nutrient metabolism, such as the brain, kidneys, pancreas and muscular tissue can be added as successive modules in order to increase the physiological relevance of the in-vitro model as we increase our understanding of metabolic communication in the system.

In a previous study we demonstrated that flow significantly affects cellular metabolism of hepatocytes, endothelial cells and adipose tissue, resulting in increased glucose uptake and an overall increase in free fatty acid (FFA) and lactate release [18]. The so-called building blocks were linked together, first in a 2-way adipose-endothelial system and then in a 3-way adipose-endothelial-hepatic connected culture [12]. In this study the 3-way culture was challenged with conditions representing normoglycaemia and hyperglycaemia by altering the composition of the common cell culture medium. The 4 media and their in-vivo equivalents were: a) Normal glucose – in which the cell culture medium contained a nominal glucose concentration of 5.5 mM representing the fasting state (FS); b) Normal glucose with insulin (65 pM) – medium simulating the post absorptive resting state (PARS); c) High glucose (20 mM) – hyperglycaemia in the absence of insulin, representing diabetes type 1 (D1); d) High glucose with 65 pM insulin – the hyperglycaemic state with inadequate glycaemic control, simulating diabetes type 2 (D2).

In all experiments an array of soluble metabolites and signaling molecules (glucose, FFA, triacylglycerides, alanine, lactate, glycerol, E-selectin, TNF-α, IL-6, albumin) was assessed by withdrawing small volumes of media from the circuit over 48 hours. The aim was to assess the capacity of this culture system to maintain/restore normal circulating metabolite concentrations while examining the effects of these conditions on other metabolites. Despite its apparent simplicity, the 3-way model shows that an imbalance of energetic substrates in the form of excess glucose, combined with insufficient insulin, changes the overall equilibrium or homeostasis of the in-vitro model and also induces general as well as specific endothelial stress, demonstrating its capacity to recapitulate salient features of systemic metabolism.

## Materials and Methods

### Cell/tissue Sources

All reagents were from Sigma-Aldrich (Sigma-Aldrich, Milan, Italy) unless otherwise specified.

Omental adipose tissue (AT) from nondiabetic subjects free of known metabolic diseases (see Ethics Statement) was treated with collagenase type II in HBSS (Hank’s Balanced Salt Solution); the partially digested tissue was placed on a 200 micron mesh filter and rinsed with DMEM (Dulbecco’s Modified Eagle Medium) to remove blood vessels. Floating partially digested AT was then divided into aliquots and transferred to DMEM with 20% FBS (Foetal Bovine Serum) ready for experiments. The partial digestion allowed us to concentrate adipocytes without destroying the collagen matrix, which is important for preserving the adipocytic phenotype [19].

Human umbilical vein endothelial cells (ECs) from Promocell (Heidelberg, Germany) were used to model the endothelium. The cells were cultured in Endothelial Cell Growth Medium (ECGM, PromoCell), composed of Endothelial Cell Basal Medium (Promocell) supplemented with 10% FBS, 0.1 ng/mL epidermal growth factor, 1.0 ng/mL basic fibroblast growth factor, 0.4% endothelial cell growth supplement/heparin, 1.0 µg/mL hydrocortisone (Promocell), 100 U/mL penicillin and 100 µg/mL streptomycin and used up to passage 4. The liver model was based on hepatocellular liver carcinoma cells (HepG2 hepatoyctes), kindly provided by Dr. S. Quarta, Laboratory of Molecular Hepatology, Department of Clinical and Experimental Medicine, University of Padua [20]. This cell line retains most of the endogenous metabolic functions of hepatocytes and was used because of its stability with respect to primary hepatocytes. HepG2 cells (HEP) were grown in Eagle’s minimal essential medium (EMEM, glucose 1 g/L) supplemented with 5% FBS, 1% nonessential amino acids, 1% EMEM vitamins, 2 mM L-glutamine, 100 U/mL penicillin and 100 µg/mL streptomycin and used up to passage 22.

### Experimental Design

To establish an in-vitro model of energetic substrate metabolism we focused on the visceral compartment of the human abdomen to determine physiologically relevant cell ratios. Cell number based allometric scaling was used to estimate the ratios of the 3 tissues in the model. Since cell numbers are proportional to organ masses and volumes, the allometric exponent for scaling cell numbers is one and cell ratios are therefore preserved when downscaling. Standard human data attributes 12% of human body mass to adipose tissue, 6.28% of body mass to vascular tissue and 2% to the liver [21]. In the internal region of the abdomen the liver occupies almost 60% (mass and volume) while adipose tissue and vascular tissue are assumed to maintain uniform distribution throughout the body. The final hepatocyte: adipocyte:endothelial cell ratio was thus estimated to be 10∶2:1. Using our previous data on cell proliferation, the number of cells added to each chamber at the beginning of the experiments was calculated to reach this ratio at the end of the 48 hour experiments [22].

**Figure 1:**
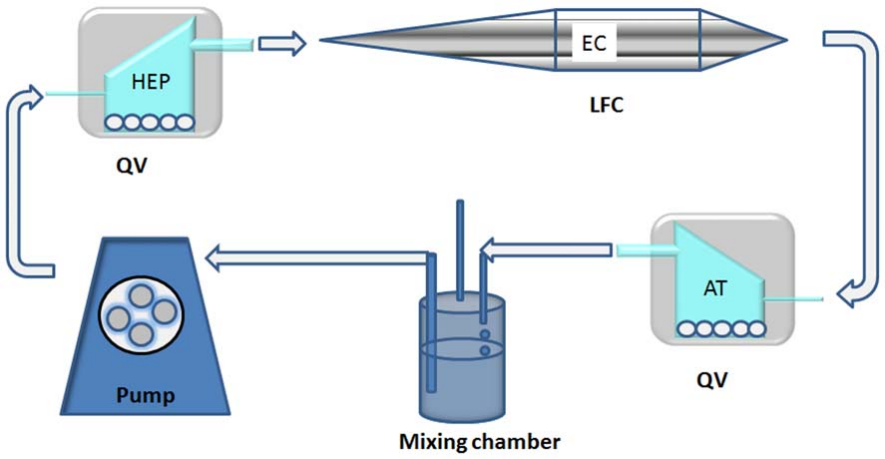
Schematic of the 3-way connected culture. QV is the low shear Quasi-Vivo chamber for hepatocytes and adipose tissue and LFC is the high shear laminar flow chamber for endothelial cells. The flow rate used was 250 µL/min. Total circuit volume is 15 mL, with 3 mL of priming volume per chamber and associated tubing and 6 mL in the mixing chamber and pump.

The in-vitro model was assembled using a modular system in which 3 different bioreactor chambers with respectively hepatocytes seeded on 3 D scaffolds, adipose tissue and endothelial cells were connected together to form a closed loop 3-way culture system. The hepatic and adipose tissue modules used were low-shear stress, high-flow inter-connectable chambers (Quasi-Vivo®, Kirkstall Ltd, Sheffield, UK), while the module used for the endothelial cell culture is a laminar flow chamber, LFC, described in ref. [23]. Fluid dynamic modelling was performed during the bioreactor design phase to estimate the shear stress in the two chambers. At the flow rate used in the experiments (250 µL/min), the wall shear stress is 0.002 Pa in the LFC and 10^−5^ Pa in the Quasi-Vivo. This flow rate was shown to be optimal for both rat and human hepatocytes [11], [22], [24]. The circuit also contains a mixing chamber for oxygenation and for the addition or sampling of media. Because media can be easily withdrawn for analysis and replenished, one of the main advantages of the inter-connected system is the possibility of conducting long term or chronic experiments in different conditions without disturbing the cells. A peristaltic pump (Ismatech IPC-4, Zurich, Switzerland) and tubing which connects the various cell chambers were also included in the circuit. The components of the bioreactor system were sterilised using H_2_0_2_ gas plasma before each use and assembled under a laminar flow hood so as to connect the three cell chambers, the pump, and the mixing chamber via tubing as shown in Figure 1.

### Cell Culture

All experiments were carried out using a common medium which had been previously tested by comparing cell vitality, morphology and function with respect to the standard media relative to each cell type [18]. The medium was established as ECGM containing 10% FBS, and in general EC were shown to be the most compromised in media other than this. Cells were incubated with normal (5.5 mM) or high (20 mM) glucose in the absence or presence of 65 pM insulin dissolved in the common medium. In order to reduce the number of variables in the experiments, other hormones known to regulate the energy balance such as glucagon (important in hypoglycaemic conditions, which were not included in the present study) and catecholamines were not included in the media.

The adipose tissue was placed in the Quasi-Vivo chamber and 1 mL medium was added to the chamber. The top of the chamber was layered with a prewetted 200 micron nylon mesh sandwiched with 2–40 micron nylon mesh circles in order to prevent movement of adipocytes out of the chamber and into the tubing [18]. The ECs were seeded onto a glass coverslip and allowed to adhere. The coverslip was then placed in the bottom of the LFC.

HEPs were seeded on collagen-coated three dimensional poly-lactide-co-glycolide scaffolds as described in [22], placed on 12 mm glass coverslips in 24 well microplates (BD Biosciences, Buccinasco, Italy) at a density of 100,000 cells per scaffold in 2 mL of ECGM. At 24 h the scaffolds were moved to new 24 multiwell. After a further 48 hours, when the cells had proliferated to about 250,000 cells per scaffold, the slides were carefully transferred to the Quasi-Vivo chamber and coated with 250 µl 1% sodium alginate dissolved in serum free medium and cross linked with 50 µl of 1% CaCl_2._ The alginate forms a thin permeable coating over the cells and acts as mechanical barrier against the tangential shear forces caused by fluid flow.

Adipose tissue, ECs, and HEPs were placed in their respective chambers and connected together in the “3-way” system. The common medium was added to the mixing chamber to bring the total medium volume up to 15 mL and the pump was turned on. When medium had filled all chambers, the flow was set to 250 µL/min after which the bioreactor was placed inside a 37°C/5% CO_2_ incubator. Medium was collected at 15, 24 and 48 H and assayed to quantify glucose, lactate, FFAs, glycerol, triacylglyceride, albumin, L-alanine, CRP (C-reactive protein), interleukin-6 (IL-6), TNF-α and E-selectin concentrations. At the end of every experiment cells/tissues were observed under a microscope to assess cell viability. Control experiments consisted of mono-cultures in static conditions, in 1-way dynamic cultures and in a 2-way AT-EC configuration, all using the same volume of media.

### Cell Viability and Metabolite Dosing

To evaluate the number of adipocytes per unit mass of tissue and confirm adipocyte viability before and after the experiments, samples of adipose tissue were further digested with 1 mg/mL collagenase in HBSS at 37°C for 30 minutes, DMEM medium containing 10% FBS was added, cells were centrifuged and floating cells were resuspended in medium. Adipocyte viability was assessed using CellTiter Blue (Promega, Italia), a colorimetric indicator of mitochondrial activity. Additionally, adipocytes were stained with Hoechst 33242 and observed using a fluorescent microscope (Olympus, AX70, Olympus Italia, Milan) to confirm nuclear integrity. There was no visible change in the size, nuclear density or in the appearance of the tissue before and after incubation and cells remained free of contamination both in the bioreactor and in static controls. EC and HEP vitality was measured using CellTiter Blue at the end of each experiment, and compared with static mono-culture controls. Cell morphology was also analysed using a microscope and the total cell number for every time point was evaluated using a Burker chamber and trypan blue to exclude non-viable cells. The number of dead cells counted was always less than 2% of the total.

Glycerol, D-lactate and L-alanine concentrations were determined by a modified Lloyd assay using an automated spectrophotometer Cobas Fara II (Roche) [25]. FFAs and glucose were measured by enzyme assays (NEFA C test-Wako Chemicals GmbH, Germany and Glucosio HK CP-HoribaABX, Italy respectively). IL-6, TNF-α, CRP, albumin and E-selectin in the medium were measured by commercial ELISA kits (Boster Biological Technology, LDT, Tema Ricerca, Bologna, Italy; Bethy Laboratories, Montgomery, TX, USA). Triacylglyceride concentrations in the medium were also assayed (Real Time, Diagnostic Systems, Viterbo, Italy), but were below the sensitivity of the kit (1 mg/dl) and were therefore not considered in our analyses. Similarly CRP and TNF-α concentrations were also below the limit of detection of the assay kits used in all the conditions tested (respectively 10 ng/mL and 1 pg/mL).

### Statistical Analysis

Statistica Version 7 was used to carry out ANOVA (Analysis of Variance), student t-tests, and Mann-Whitney tests. Data were expressed as the mean ± sd (standard deviation). All experiments were carried out at least in triplicate, and the dosing was performed in triplicate. Variations were considered to be significant if the p-value was <0.05. Unless noted, the p-values in the text refer to those obtained from student t-tests. Mann-Whitney and Kruskal-Wallis analyses confirmed those obtained with t-tests in all cases.

### Ethics Statement

Omental adipose tissue was obtained, with written and informed consent, from surgical interventions in nondiabetic subjects free of known metabolic disease. Samples were received and analysed anonymously. The study was approved by the Local Ethical Committee of the Local Hospital Agency of Padua (Comitato Etico, Azienda Ospedaliera di Padova).

**Figure 2:**
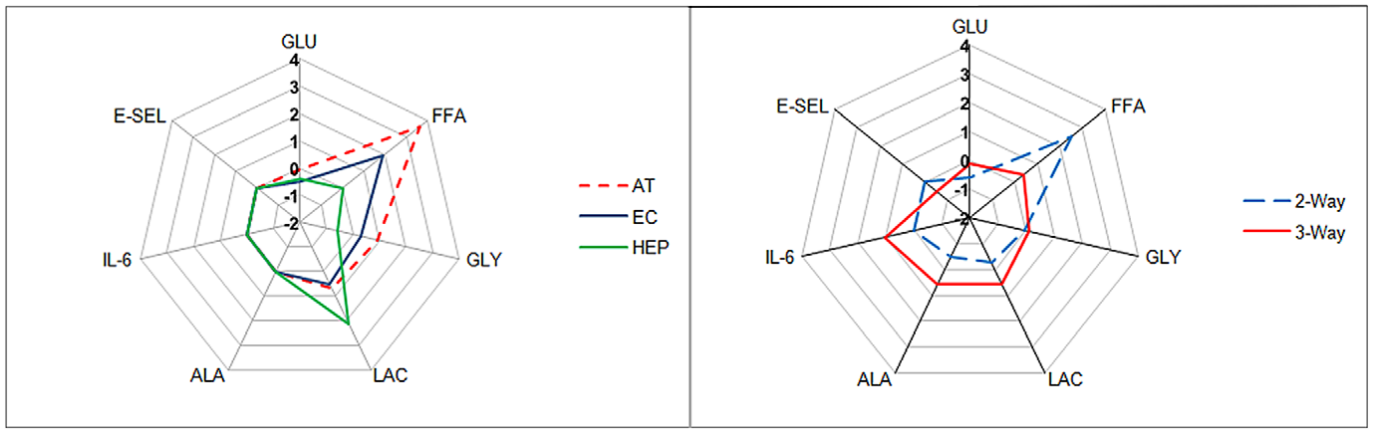
Star plot of measured metabolites in fasting state conditions at 48 H. A) Fractional variation in metabolite concentrations for 1-way dynamic cultures of AT, EC and HEP (data published in ref. [18]). B) Fractional variation in metabolite concentrations for 2-way (AT+EC) connected culture and 3-way (AT+EC+HEP) connected culture. GLU (glucose), GLY (glycerol), LAC (lactate), ALA (L-alanine), E-SEL (E-selectin) (data partially published in ref [12]).

**Figure 3:**
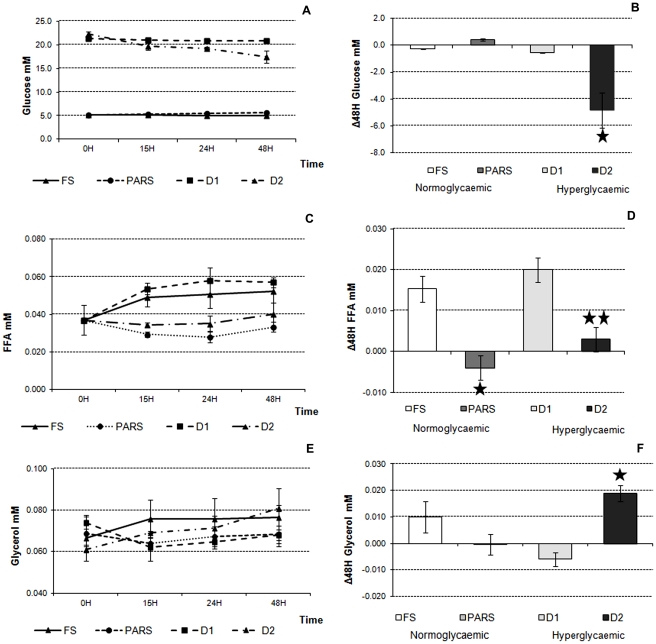
Glucose, FFA and glycerol in the 3-way cultures in FS, PARS, D1 and D2 conditions. A) Glucose variations over time. B) Changes in medium glucose concentration with respect to initial media concentration at 48 H (* = p<0.05 vs. D1). C) FFA variations over time. D) Changes in medium FFA concentration with respect to initial media concentration at 48 H (* = p<0.05 vs. FS; ** = p<0.05 vs. D1). E) Glycerol variations over time. F) Changes in medium glycerol concentration with respect to initial media concentration at 48 H (* = p<0.05 vs. D1). In all cases time 0 represents the levels of metabolites in fresh media. Data are expressed as means ± SD (3≤n≤6). Error bars represent the standard deviation.

## Results

### The 3-way Connected Culture in FS Conditions

The 3-way connected culture of hepatocytes, endothelial cells and adipose tissue is a simplified in-vitro model of endogenous metabolism in the visceral region, which resembles the in-vivo state more closely than traditional cell culture models. Before culturing the 3 tissues together in the 4 different conditions, metabolite dynamics and cell viability in static cultures, 1-way cultures and 2-way (adipose tissue and endothelial cells) cultures were investigated over a period of 48 hours in the FS condition, with normal glucose media. For all three cells/tissue, an increase of viability was observed with respect to static mono-culture controls (% increases with respect to controls: AT 10±3%, p<0.05, EC 50±5% p<0.004, HEP 25±3% p<0.001), consistent with our previous experiments comparing static mono-cultures, static co-cultures and 2- and 3-way connected cultures [12], [18], [26], [27]. As discussed therein, the increase in viability in the 3-way system can be attributed to an increased rate of nutrient turnover and oxygen supply, an interstitial-like shear stress due to flow and the presence of cell crosstalk.

The 1, 2 and 3-way metabolite data are summarized in Figure 2 in the form of star plots which represent the fractional change in metabolite concentration with respect to the initial normal glucose media concentrations after 48 H (Δ/0 H value). The 1- and 2-way cultures were unable to maintain stable glucose, FFA, lactate and glycerol levels over time. In the 3-way connection, glucose, glycerol and FFA levels did not change over 48 H, while albumin synthesis and lactate and L-alanine release were increased with respect to the 2-way system.

### Glucose Dynamics

As shown in Fig. 3A, glucose remained stable over time in the experiments using normal glucose (FS and PARS) and we did not observe any effect of insulin on cellular glucose uptake in these states. On the contrary, at 20 mM glucose, insulin was able to increase glucose uptake steadily over 48 H, but glucose concentrations still remained high after 48 H, dropping by−4.8 ± 1.3 mM over 48 H in D2 vs. 0.435 ± 0.085 mM in D1 (Fig. 3B). This observation suggests that whilst the 3-way system is adequate in reversing hypoglycaemia, as shown in Fig. 2B, it is not as effective in the response to hyperglycaemia, at least in this experimental setting. In the D1 condition, the response of the system is similar to what is observed in type 1 diabetics who are unable to synthesize insulin in response to hyperglycaemia. The addition of insulin to the system, simulating diabetes type 2, improved glucose disposal significantly but was not sufficient to restore normal glucose concentrations, probably because the insulin dose was quite small, equivalent to a hypoinsulinemic state, suggesting that the high levels of glucose provoke a sort of “insulin resistance” in-vitro. Desensitization to insulin in the presence of hyperglycaemia in-vitro has been widely documented for many cell types [28], [29] and has also been shown in co-cultures of adipocytes and hepatocytes [30], [31].

### FFA and Glycerol Dynamics

In the absence of insulin (FS and D1), FFA was released into the medium. Insulin significantly (p<0.05) blunted FFA release into the culture medium in both PARS and D2 conditions (Figs. 3C and 3D).

Figs. 3E and 3F show a slight glycerol release into the culture medium over time (0.010±0.016 mM in 48 H) in the FS condition. The addition of insulin, simulating the post absorptive resting state, prevented this increase. This trend mirrors that of FFAs. The FS condition is likely characterized by some baseline lipolysis, which can be blocked by the addition of insulin. On the other hand we observed a significant decrease in medium glycerol (−0.006±0.003 mM in 48 H, p<0.05) in D1, in contrast to what was observed with FFAs. When insulin was added, extracellular glycerol increased two-fold (0.019±0.003 mM in 48 H). This also contrasts with the trend of FFAs and demonstrates that high glucose has divergent effects on FFA and glycerol release, which can be modulated by the addition of insulin.

### Lactate

**Figure 4:**
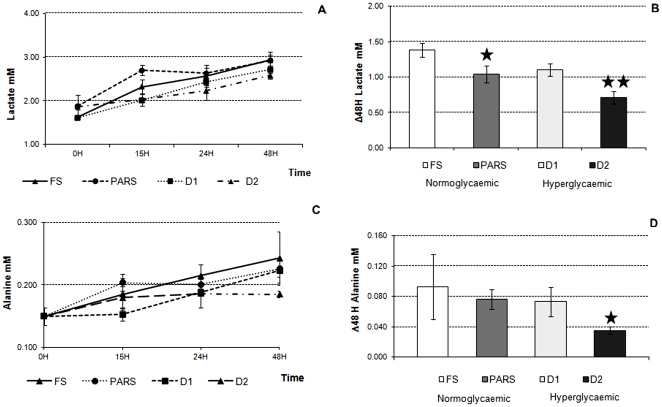
Medium lactate and L-alanine in the 3-way cultures in FS, PARS, D1 and D2 conditions. A) Lactate variations over time. B) Changes in medium lactate concentration with respect to initial media concentration at 48 H (* = p<0.05 vs. FS; ** = p<0.05 vs. D1). C) L-alanine variations over time. D) Changes in medium L-alanine concentration with respect to initial media concentration at 48 H (* = p<0.01 vs. D1). In all cases time 0 represents the levels of metabolites in fresh media. Data are expressed as means ± SD (3≤n≤6). Error bars represent the standard deviation.

There was a net increase in medium lactate for all four conditions (Fig. 4A), with the FS condition showing the highest cellular lactate release of 1.386±0.099 mM over 48 H. The addition of insulin in the PARS resulted in a lower lactate release of 1.039±0.120 mM over 48 H. A lower level of lactate release was observed in D1 (1.104±0.090 mM over 48 H) with respect to the fasting state condition. And finally, the addition of insulin to the high glucose medium resulted in the smallest lactate release observed, 0.711±0.088 mM over 48 H (Fig. 4B). A Pearson’s Correlation of −0.96 (p<0.05) shows that higher glucose concentrations correlate with less lactate release suggesting that changes in the medium glucose concentration can largely explain total lactate release.

### Albumin and Alanine

Albumin is an important marker of hepatic function. Albumin concentrations were similar in all 4 conditions and consistently higher than in the 1-way HepG2 monoculture (12.6±1.2 nM vs. 2.4±0.2 nM over 48 H, p<0.0001) indicating increased albumin synthesis and enhanced hepatocyte function in the 3-way system [18].

A net increase in L-alanine levels was observed over time in all media (Fig. 4C). In FS, PARS and D1 conditions, similar quantities of L-alanine were released in the medium. However in D2, when insulin was added to the high glucose media, L-alanine levels were almost halved (0.073±0.002 mM in D1 vs. 0.035 ± 0.005 mM in D2; p =  0.0195) (Fig. 4D). This suggests that the presence of insulin in the hyperglycaemic media determined increased amino acid uptake with respect to the other media.

### Cytokines and E-selectin

**Figure 5:**
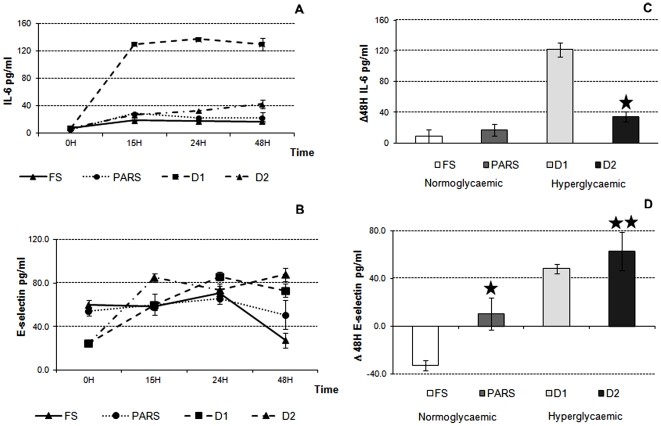
Medium IL-6 and E-selectin **in the 3-way cultures in FS, PARS, D1 and D2 conditions.** A) IL-6 variations over time. B) Summary of the changes in Il-6 concentrations over 48 H (* = p<0.05 vs. D1). C) E-selectin variations over time. D) Summary of the changes in E-selectin concentrations over 48 H (* = p<0.05 vs. FS, ** = p<0.05 vs. D1). Time 0 represents the levels of markers in fresh media. Data are expressed as means ± SD (3≤n≤6). Error bars represent the standard deviation.

There was a slight increase in IL-6 levels over time in the FS condition, and this trend was similar in the PARS (25.8 ± 8.7 pg/mL in FS and 34.5 ± 7.8 pg/mL in PARS over 48 H) (Fig. 5A). In D1 a net increase in IL-6 was observed at 15 H (from 8.3 ± 0.2 to 130 ± 9.0 pg/mL, p<0.001). Then, cytokine levels remained stable over time with a IL-6 release in the medium of 121.7 ± 6.6 pg/mL (Fig. 5B). The addition of insulin in D2 significantly reduced IL-6 levels in the medium over 48 H (34.6 ± 6.6, p<0.0001). Reference IL-6 levels in serum are in the range 0–25 pg/mL, while higher serum values are characteristic of an inflammatory status.

As shown in Fig. 5C, in the first 24 H, E-selectin concentrations did not change significantly under normal glucose media, both in the FS and PARS conditions. Then, in the absence of insulin, E-selectin levels dropped at 48 H, while they remained stable in the PARS (Fig. 5D). Under high glucose concentrations, in both states D1 and D2, E-selectin increased significantly within the first 15 H (p<0.009) and then remained stable. The expression of E-selectin did not change appreciably with the addition of insulin in D2, indicating that endothelial stress is due to the high glucose content of the medium. It should be noted that the levels of E-selectin detected were within the lower limits of the assay, and certainly much lower than those typically reported for activated ECs [32], [33].

## Discussion

### Insulin and Glucose Modulation of Metabolite Dynamics: Parallels with In-vivo States

The data in Figure 2 summarise the differences between 1, 2 and 3 way cultures. When exposed to FS conditions, the 2-way connected culture of adipose tissue and endothelial cells resulted in dropping glucose and rising FFA concentrations over time. The addition of hepatocytes to the culture, forming a 3-way system, was able to restore normal glucose levels and remove FFAs from the circulating medium, much as hepatocytes are able to prevent hypoglycaemia in-vivo by responding to dropping glucose concentrations with gluconeogenesis [12], [18]. When the 3-way connected culture was exposed to a low dose of insulin with normal glucose, simulating the post adsorptive resting state, medium FFA and glycerol decreased over 48 H, suggesting inhibition of lipolysis, much as lipolysis is inhibited post-prandially in-vivo, although increased HepG2 hepatocyte FFA uptake cannot be excluded [34], [35]. In fact insulin significantly reduces FFA release at both normal and high glucose conditions as observed in-vivo. In the absence of insulin, high glucose may promote adipose tissue lipolysis and simultaneously inhibit hepatic FFA uptake [36], [37]. The observation of increased FFAs with high glucose in the absence of insulin, a condition not usually seen in-vivo, nonetheless brings attention to the fact that hyperglycaemia, in the absence of adequate insulin, can contribute to high circulating FFAs, which are associated with other morbidities such as obesity [38], cardiopathy [39], and atherothrombosis [40].

In contrast to the FFA trend, in the model, insulin had a glucose-dependent effect on glycerol release. Medium glycerol levels were reduced in normal glucose media (PARS) and increased in high glucose media (D2 state) by insulin. Previous reports on perfused rat adipocytes have shown that insulin can inhibit FFA release while increasing glycerol release and that lipolytic oscillations are extremely sensitive to glucose and insulin concentrations [41].

In humans, lactate levels usually rise after feeding and drop after fasting. There was net lactate release over 48 H for all conditions tested, with the D2 state causing a reduction in lactate release. In the 3-way connection, in FS conditions the observation of lower lactate release compared with individual monocultures (Figure 2 and ref. [18]), probably represents some hepatic lactate retention to serve as a precursor for gluconeogenesis, followed by glucose release to replace glucose removed from the medium by ECs and adipose tissue. In D2, hepatic lactate retention may also fuel gluconeogenesis, but in this case followed by glycogenesis rather than glucose release given that extracellular glucose concentrations remain high despite improved glucose disposal with insulin, which is known to increase hepatic glycogen synthesis [42].

L-alanine is a precursor of gluconeogenesis and it plays an important role in maintaining the body’s blood glucose balance. It is a nonessential amino acid derived from pyruvate transamination. In the liver alanine is converted back to pyruvate which is a source for gluconeogenesis. An increase in L-alanine levels was observed under all four different experimental conditions over 48 H. This may be due to an increase of amino acid synthesis and/or the conversion of pyruvate by hepatic cells. In normal glucose media, L-alanine production over 48 H was slightly decreased by insulin, indicating an increase of protein anabolism. Inhibition of protein catabolism, possibly associated with increased protein synthesis, was quite significant (p>0.01) in D2 conditions, likely due to the action of insulin triggered by high glucose levels.

### IL-6 and E-selectin Concentrations are Increased in the Presence of a Hyperglycaemic Media

Although we did not detect appreciable levels of TNF-α and CRP in any of our experiments, both IL-6 and E-selectin were increased in D1 and D2 conditions. In-vivo, muscle-derived IL-6 is released into the circulation and exerts a glucoregulatory effect on the liver and adipose tissue. It is involved in other metabolic pathways, since with the increase in liver glucose output during IL-6 infusion, Stouthard et al. [43] observed an increased release of FFAs. Furthermore, infusion of IL-6 into rats increased serum triacylglyceride and FFA levels in a dose-dependent manner [44]. IL-6 production has also been observed in human adipocytes [45]. In fact, IL-6 was released in the medium in all four different conditions, but its levels were enhanced in the D1 condition. Here FFA levels were increased and glucose levels were maintained high in the medium. This suggests that the IL-6 release mirrors an oxidative stress and/or inflammation condition of the system caused by high glucose. The addition of insulin in D2 determined a drop of glucose and FFAs and in turn a drop of IL-6 levels. The increase of medium IL-6 release seems to be modulated through high glucose levels rather than FFAs, since IL-6 levels were comparable and independent of insulin and FFA content in FS and PARS conditions.

E-selectin is synthesized and expressed only by activated endothelial cells and not by other cell types and its presence in serum should reflect the state of endothelial damage and regeneration [46]. In our experimental model, we observe an immediate and consistent increase of E-selectin in the medium in both D1 and D2 conditions. On the other hand, the normal glucose concentrations do not induce increased E-selectin release into the medium, showing that, hypergylcemic conditions can cause endothelial stress as demonstrated both in-vivo and in-vitro [47], [48].

**Figure 6:**
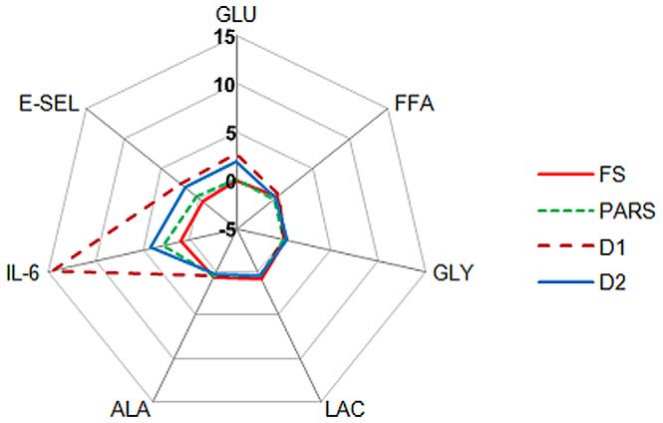
Star plot of measured metabolites in the 3-way connected cultures. Fractional variation in GLU (glucose), FFA, GLY (glycerol), LAC (lactate), ALA (L-alanine), IL-6 and E-SEL (E-selectin) at 48 H in fasting, post-adsorptive resting state, diabetes 1 and diabetes 2 simulating conditions in the 3-way connected culture.

The most significant results can be summarized in star plot representing the fractional variation in metabolite or proinflammatory marker concentration in the medium at 48 H with respect to the basal medium concentration (Δ/0 H value). Figure 6 clearly illustrates how an imbalance of energetic substrates, in the form of excess glucose, combined with insufficient insulin, changes the overall equilibrium or homeostasis of the in-vitro model system and also induces general as well as specific endothelial stress. Furthermore, the plot highlights that while small quantities of insulin are sufficient to contain “systemic” stress levels and FFA circulation in the medium, these hormone levels have little or no influence on endothelial injury.

This is the first example of an in-vitro model of endogenous metabolism which analyses the network of interactions between different cell types. Despite its apparent simplicity, the 3-way model has enormous potential as a tool to study the comprehensive effects of different tissues in integrated metabolism. By connecting three metabolically relevant tissues together and analyzing metabolite dynamics in different conditions, we simulated 4 different physiological and pathological conditions including the fasting state, the post adsorptive state and the post prandial state in type 1 and type 2 diabetes, respectively. The results demonstrate that a properly scaled connected culture of adipose tissue, endothelial cells and hepatocytes can recapitulate some of the features of human metabolism such as systemic inflammation in the presence of nutritional overload.
